# Sintilimab combined with apatinib plus capecitabine in the treatment of unresectable hepatocellular carcinoma: A prospective, open-label, single-arm, phase II clinical study

**DOI:** 10.3389/fimmu.2022.944062

**Published:** 2022-08-26

**Authors:** Dailong Li, Lu Xu, Jinxing Ji, Dan Bao, Juan Hu, Ying Qian, Yinjie Zhou, Zhuo Chen, Daojun Li, Xiaopeng Li, Xiaoling Zhang, Hao Wang, Changjun Yi, Menglu Shi, Yaqi Pang, Siqi Liu, Xinhua Xu

**Affiliations:** ^1^ Department of Oncology, Yichang Central People's Hospital and The First College of Clinical Medical Science, China Three Gorges University, Yichang, China; ^2^ Tumor Prevention and Treatment Center of Three Gorges University and Cancer Research Institute of Three Gorges University, Yichang, China; ^3^ Department of Radiation Oncology and Medical Oncology, Zhongnan Hospital of Wuhan University, Wuhan, China; ^4^ Yichang Akeman Pathology and Diagnostics Center, Yichang, China

**Keywords:** apatinib, capecitabine, first-line treatment, hepatocellular carcinoma, immune checkpoint inhibitors, sintilimab

## Abstract

**Objective:**

To evaluate the efficacy and safety of sintilimab combined with apatinib plus capecitabine in the treatment of unresectable hepatocellular carcinoma (HCC) to provide a more effective first-line treatment for patients with advanced HCC.

**Methods:**

This open-label, prospective, phase II study included patients with unresectable HCC who did not receive systematic treatment. The patients were treated with sintilimab (200 mg, intravenous drip, once every 3 weeks) combined with apatinib (250 mg, oral administration, once a day) plus capecitabine (1000 mg/m^2^, twice a day; after 2 weeks of oral administration, the drug was stopped for 1 week; course of treatment, 3 weeks). The primary endpoint was the objective response rate (ORR). The secondary endpoints included disease control rate (DCR), progression-free survival (PFS), duration of response (DoR), overall survival (OS), and safety.

**Results:**

Forty-seven patients (1 lost to follow-up) were enrolled in the study. As of March 1, 2022, the ORR and DCR were 50.0% (95% CI: 34.9–65.1%) and 91.3% (95% CI: 79.2–97.6%), respectively, after blind, independent imaging evaluation. The median follow-up time was 18.7 months (95% CI: 17.2–20.2 months). The median PFS was 9.0 months (95% CI: 7.1–10.9 months). The median DoR was 10.8 months (95% CI: 4.8–16.8 months). The median OS was not reached, and the 1-year OS rate was 71.7% (95% CI: 56.5–84.0%). Only 28.3% (13/46) of patients had grade 3/4 treatment-related adverse events.

**Conclusion:**

Sintilimab combined with apatinib plus capecitabine has good safety and anti-tumor activity as a first-line treatment for unresectable HCC. This is worthy of further multi-center, prospective, randomized, large-sample clinical studies.

**Clinical Trial Registration:**

https://ClinicalTrials.gov, identifier NCT04411706.

## Introduction

According to the annual statistics of GLOBOCAN2020, of all cancers, liver cancer has the sixth highest incidence and the third highest mortality globally (1). Primary liver cancer mainly includes three different pathological types: hepatocellular carcinoma (HCC), intrahepatic cholangiocarcinoma (ICC), and the HCC-ICC mixed type, of which HCC accounts for 90% (2). Liver cancer often has no obvious clinical symptoms in the early stages, and the condition is commonly advanced at the time of treatment initiation when it is not suitable for surgery or local treatment. The 5-year survival rate of patients with unresectable HCC is less than 15% (3, 4).

At present, the main treatment methods are systemic therapies, including molecular-targeted therapy, chemotherapy, and immunotherapy. Sorafenib is a representative drug for molecular-targeted therapy of unresectable HCC. In the SHARP and Oriental studies that established the status of sorafenib as the first-line treatment for unresectable HCC, the objective response rate (ORR) to sorafenib was only approximately 3%, and the median overall survival (OS) was only barely more than 2 months longer than that of the placebo group (5, 6). The results of a randomized controlled international multi-center phase III clinical study showed that the ORR of the FOLFOX4 regimen reached 8.15% (7). However, the adverse reactions to chemotherapy were relatively severe, making it unsuitable as the mainstream treatment for unresectable HCC.

The advent of immunotherapy brings new possibilities and hope for treating unresectable HCC. According to the results of CheckMate-040 (8) and KEYNOTE-224 (9), the National Comprehensive Cancer Network (NCCN) and Chinese Society of Clinical Oncology guidelines recommend using nivolumab and pembrolizumab (PD-1 inhibitors) as second-line treatment options for advanced HCC. However, the recent CheckMate-459 study showed that the median OS of nivolumab and sorafenib in the first-line treatment of unresectable HCC patients was 16.4 months and 14.7 months, respectively (hazard ratio [HR] = 0.85, P = 0.0752); the primary endpoint OS did not reach the pre-set threshold of statistical significance (HR = 0.84; P = 0.0419), while progression-free survival (PFS) was 3.7 months and 3.8 months, respectively (10). Similarly, the KEYNOTE-240 study showed that the median OS, PFS, and ORR of pembrolizumab versus placebo as a second-line treatment for unresectable HCC was 13.9 months and 10.6 months, 3.0 months and 2.8 months, and 18.3% and 4.4%, respectively. This clinical trial did not meet the pre-set statistical requirements for OS or PFS (11).

Although targeted therapy and immunotherapy have achieved good results for unresectable HCC, the clinical effects of single drugs are limited. In recent years, some clinical studies have shown that combination therapy is a future development direction for unresectable HCC. For example, immunotherapy combined with anti-angiogenic drugs (camrelizumab plus apatinib) (12), double immunotherapy (nivolumab plus ipilimumab) (13), and immunotherapy combined with chemotherapy (camrelizumab plus FOLFOX4) (14) have yielded good results. In particular, a global phase III randomized controlled trial, the IMbrave150 study (15), showed that the efficacy of atezolizumab combined with bevacizumab in the treatment of unresectable HCC was significantly better than that of sorafenib; this combination became the first FDA-approved first-line treatment, based on immunotherapy, for unresectable HCC. The IMbrave150 study has greatly encouraged research regarding the best combination for treating unresectable HCC.

Sintilimab is a highly selective all-human PD-1 monoclonal antibody that has shown excellent efficacy and safety in the treatment of relapsed or refractory classical Hodgkin lymphoma. A recent multi-center randomized controlled trial showed that sintilimab plus a bevacizumab biosimilar (IBI305) had significantly better efficacy than sorafenib in the treatment of unresectable HCC, with acceptable safety (16). As an inhibitor of vascular endothelial growth factor receptor (VEGFR), apatinib can inhibit tumor angiogenesis and exert anti-tumor effects (17, 18). A phase III clinical study has confirmed that apatinib significantly prolonged the median PFS and OS (4.5 *vs*. 1.9 months; 8.7 *vs*. 6.8 months; P < 0.05) in patients with advanced HCC, compared with the placebo treatment (19). Capecitabine is a new 5-FU prodrug developed by Roche (Basel, Switzerland). It is rapidly absorbed by the intestinal mucosa after oral administration and is transformed into 5-FU by catalytic action in the human liver and tumor tissue, thus playing an anti-tumor role. Therefore, capecitabine has a strong targeting effect in advanced liver cancer (20). In addition, after oral administration, the concentration of transformed 5-FU in the tumor tissue is high, which can improve the curative effect and reduce adverse reactions, and the compliance of patients with oral administration of the drug is good. A multi-center study showed that oral capecitabine is a safe and effective option for patients with HCC for which targeted and other treatments are unsuitable (21).

Increasing evidence has shown that chemotherapy has the potential to overcome immunosuppression (22, 23), promote tumor antigen presentation (24, 25), and regulate anti-vascular activity (26, 27). Chemotherapy combined with PD-1/L1 and VEGFR inhibitors may have a synergistic effect. A phase III clinical study of atezolizumab (anti-PD-L1 antibody) combined with bevacizumab (anti-VEGF antibody) plus chemotherapy in the treatment of non-small cell lung cancer confirmed that this regimen prolongs the survival time of patients (28), providing evidence for this combined strategy. However, to date, there have been no reports on the combination of immune checkpoint inhibitors with anti-angiogenic agents and chemotherapy for treating patients with unresectable HCC.

Therefore, this study aimed to evaluate the efficacy and safety of sintilimab combined with apatinib plus capecitabine in treating unresectable HCC prospectively and provide a more effective first-line treatment for patients with unresectable HCC.

## Materials and methods

### Determination of sample size

Simon’s two-stage design was used to estimate the sample size of the primary endpoint. The bilateral test level α was 5%, and the test efficiency 1-β was 80%. According to the available data, the ORR of sorafenib or lenvatinib as first-line treatment for unresectable HCC is between 9% and 24%. The ORRs of camrelizumab (PD-1 inhibitor) combined with apatinib and camrelizumab combined with chemotherapy (FOLFOX4) as first-line treatment for unresectable HCC were 30.8% and 27.3%, respectively. Capecitabine is a well-tolerated and effective chemotherapeutic agent for patients with HCC. We estimated that the ORR of sintilimab combined with apatinib and capecitabine was 50%. Based on this assumption, we planned to enroll 15 patients in the first stage, and if the treatment was considered effective in five patients (assessed as a complete or partial response) in the first stage, we would move on to the second stage, with 46 patients in total in the two stages. Overall, if 23 or more patients achieved partial (PR) or complete response (CR), the treatment regimen would be considered a better clinical strategy.

### Study design and patients

This prospective single-arm phase II clinical trial was approved by the Medical Ethics Committee of Yichang Central People’s Hospital and was conducted in accordance with the tenets of the Declaration of Helsinki. This study was registered at ClinicalTrials.gov (NCT04411706). Written informed consent was obtained from all patients.

Patients were deemed eligible if they met the following criteria (1): HCC confirmed by pathology, considered unsuitable for surgery or other local treatment, and Barcelona clinical HCC (BCLC) stage B or C (2); age between 18 and 75 years (3); Eastern Cooperative Oncology Group (ECOG) performance status of 0 or 1 (4); at least one measurable lesion (according to the Response Evaluation Criteria in Solid Tumors v1.1 (RECIST v1.1)) (5); at least 3 months of expected survival time (6); liver function of Child-Pugh class A or B (6); main organ functions meeting the following criteria (no blood components and cell growth factors were used during the screening period): absolute neutrophil count ≥ 1.5 × 10^9^/L, platelets ≥ 80 × 10^9^/L, hemoglobin ≥ 90 g/L, serum albumin ≥ 30 g/L, alanine aminotransferase and aspartate aminotransaminase ≤ 2.5 × upper limit of normal [ULN], total bilirubin ≤ 1.5 × ULN, alkaline phosphatase ≤ 2.5 × ULN, serum creatinine ≤ 1.5 × ULN, and thyroid-stimulating hormone (TSH) ≤ 1 × ULN (both free triiodothyronine [FT3] and free thyroxine [FT4] levels should be measured, and subjects can be enrolled if FT3 and FT4 levels are normal); and (7) chronic hepatitis C virus (HCV) or hepatitis B virus (HBV) infection (viral load < 500 IU/ml before enrollment).

The exclusion criteria were as follows (1): receiving other anticancer treatments, including immunotherapy, targeted therapy, and chemotherapy (2); any history of active autoimmune disease, concurrent medical use of immunosuppressive medications, or immunosuppressive doses of systemic corticosteroids (3); central nervous system metastasis or hepatic encephalopathy, liver transplantation recipients, hypertension not controlled by antihypertensive drugs, and clinically obvious cardio-cerebrovascular diseases (including congestive heart failure, uncontrolled arrhythmia, angina requiring long-term medical therapy, valvular heart disease, and myocardial infarction, as well as transient ischemic attack, cerebral hemorrhage, cerebral infarction, pulmonary embolism and deep venous thrombosis occurring within 6 months before enrollment) (4); history of gastrointestinal bleeding or obvious tendency for gastrointestinal bleeding within the previous 3 months (5); past allergy to biological agents (6); other active malignant tumors.

### Procedures

All patients were treated with sintilimab combined with apatinib and capecitabine. Sintilimab injection (200 mg, diluted in 100 mL normal saline) was administered intravenously over 30–60 min. This was the first day of a 21-day cycle. Capecitabine tablets 1000 mg/m^2^ were administered orally twice a day from days 1 to 14. After 2 weeks, the drug was discontinued for 1 week. The treatment cycle was 21 days. Apatinib 250 mg was administered orally once a day. The dose of sintilimab was fixed, and dose adjustment was not allowed. Dose adjustments were allowed for apatinib and capecitabine. Three-drug combinations of two or more cycles were defined as effective cases. If apatinib or capecitabine could not be tolerated after two cycles, immunotherapy alone was allowed to continue until disease progression or intolerance occurred.

Effectiveness was evaluated by researchers according to the RECIST v1.1 guidelines. The baseline assessment was completed within 7 days before the first administration. During the study, imaging evaluation was performed every two cycles according to the RECIST v1.1 guidelines until disease progression, death, or the end of the study, whichever occurred first.

Data of adverse events (AE) and laboratory abnormalities were collected from the beginning of the assigned treatment to 30 days after cessation of treatment. Severe adverse reactions and immune-related adverse events (irAE) were monitored for 90 days. Adverse events and laboratory results were graded according to the Common Terminology Criteria for Adverse Events (CTCAE v5.0) (29).

All patients were followed up after the first treatment administration and once a month after completion of treatment until disease progression, death, loss to follow-up, other anti-tumor therapy, or research termination. Patients who stopped treatment for reasons other than imaging progression, such as adverse reactions, needed to be followed up for tumor progression and were evaluated as planned. All patients were followed up until death or for not less than 1 year.

After patients signed an informed consent form, three unstained tumor tissue sections were obtained from each patient during the screening period. The expression level of PD-L1 was detected by PD-L1 IHC 28-8 pharmDx (Dako Products, Santa Clara, CA, USA). The expression of PD-L1 was determined by the tumor proportion score (TPS): TPS = (the number of tumor cells positive for PD-L1 staining/the total number of live tumor cells in the sample) × 100%.

### Outcomes

The primary endpoint was the ORR, defined as the percentage of patients assessed as showing a complete or partial response by blind, independent image analysis, according to the RECIST v1.1 guidelines. The secondary endpoints were safety, disease control rate (DCR; percentage of patients with CR, PR, or stable disease [SD] as the best overall response), progression-free survival (PFS; time from the first dose of the study drug to disease progression or death from any cause), duration of response (DoR; duration until first confirmed partial or complete response to disease progression or death), and OS (the time from the first dose of the study drug to death from any cause). The correlation between PD-L1 expression and patient survival was also investigated.

### Statistical analysis

The Kaplan–Meier method was used to analyze the time-event variables, and the log-rank test was used to analyze the differences in PD-L1 expression. Safety was described in terms of number and percentage, using descriptive statistics. The ORR and DCR with corresponding 95% confidence intervals (CI) were calculated using the Clopper–Pearson method. All statistical tests were two-sided, with the level of significance set at 0.05. All statistical analyses were performed using SPSS software (version 26.0; IBM, Armonk, NY, USA) and R software (version 4.1.1; R Foundation, Vienna, Austria).

## Results

### Patient baseline characteristics

**Figure 1:**
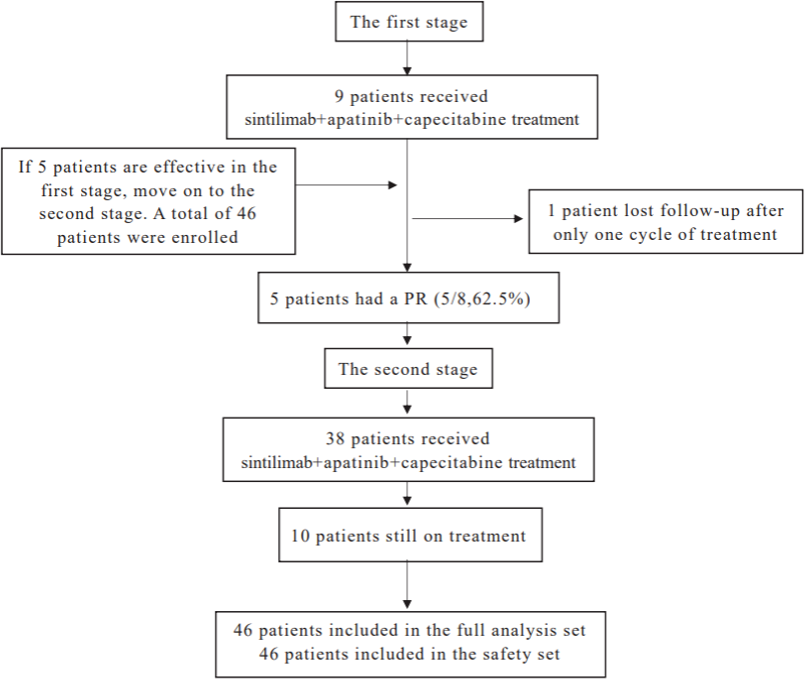
Trial flow diagram.

From June 2020 to March 2021, 47 patients with unresectable HCC were enrolled and treated with sintilimab combined with apatinib plus capecitabine. In the first stage, one patient was lost to follow-up after only one treatment cycle. Forty-six patients were included in the safety analysis and in the full analysis set (**Figure 1**). The baseline characteristics of the 46 patients included in this analysis are shown in **Table 1**. The median age of the patients was 56 years (range: 30–75 years); 41 patients (89.1%) had BCLC stage C, 29 (63.0%) had hepatitis B virus infection, 34 (73.9%) had extrahepatic spread, 14 (30.4%) had portal vein invasion, and 30 (65.2%) had an ECOG score of 1. There were 35 patients (76.1%) with a PD-L1 TPS < 1%, while 11 patients (23.9%) had a PD-L1 TPS ≥ 1%.

**Table 1:** Baseline clinical characteristics.

Characteristic	Sintilimab+apatinib+capecitabine
	All patients (N = 46)
**Median age (range)**	56 (30-75)
**Gender**—**No. (%)**	
Male	39 (84.7)
Female	7 (15.3)
**ECOG PS—No. (%)**	
0	16 (34.8)
1	30 (65.2)
**BCLC—No. (%)**	
B	5 (10.9)
C	41 (89.1)
Pathological differentiation^a^—No. (%)	
III	9 (19.6)
IV	35 (76.1)
Unknown	2 (4.3)
**HBV infection—No. (%)**	
Yes	29 (63.0)
No	17 (37.0)
**Child-Pugh class—No. (%)**	
A	37 (80.4)
B	9 (19.6)
**Extrahepatic spread—No. (%)**	
Yes	34 (73.9)
No	12 (26.1)
**Portal vein invasion—No. (%)**	
Yes	14 (30.4)
No	32 (69.6)
**AFP, ng/ml—No. (%)**	
<400	24 (52.2)
≥400	22 (47.8)
**PD-L1 TPS—No. (%)**	
<1%	35 (76.1)
≥1%	11 (23.9)

a Pathological differentiation was based on the Edmondson-Steiner grading method for hepatocellular carcinoma.

### Efficacy

**Figure 2:**
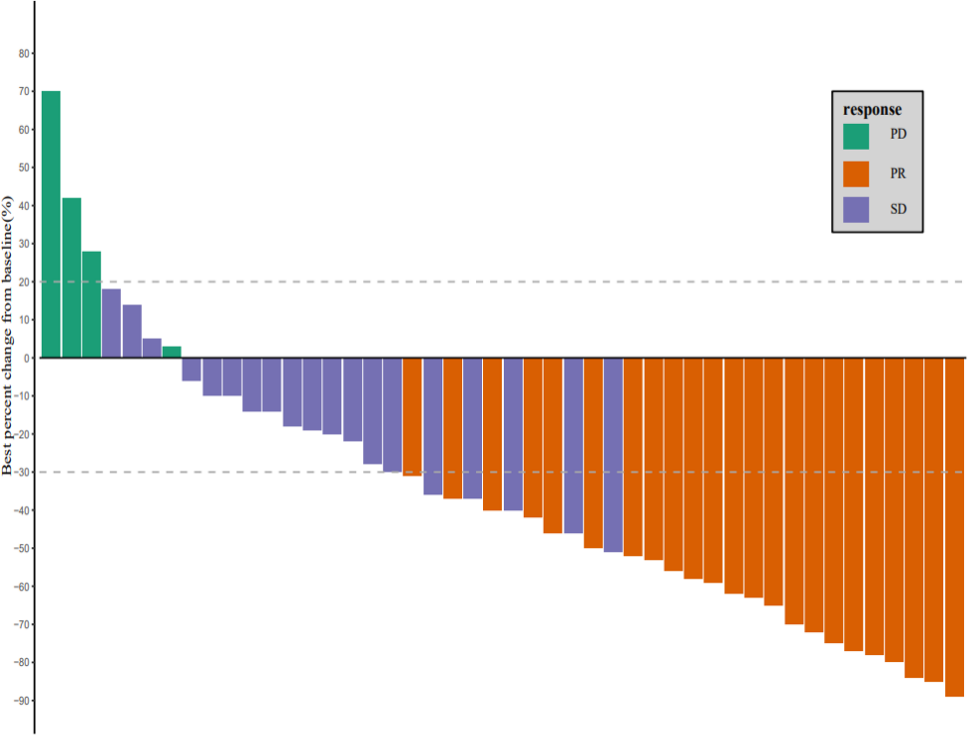
Waterfall plots of best changes in the size of the target lesion versus baseline.

**Figure 3:**
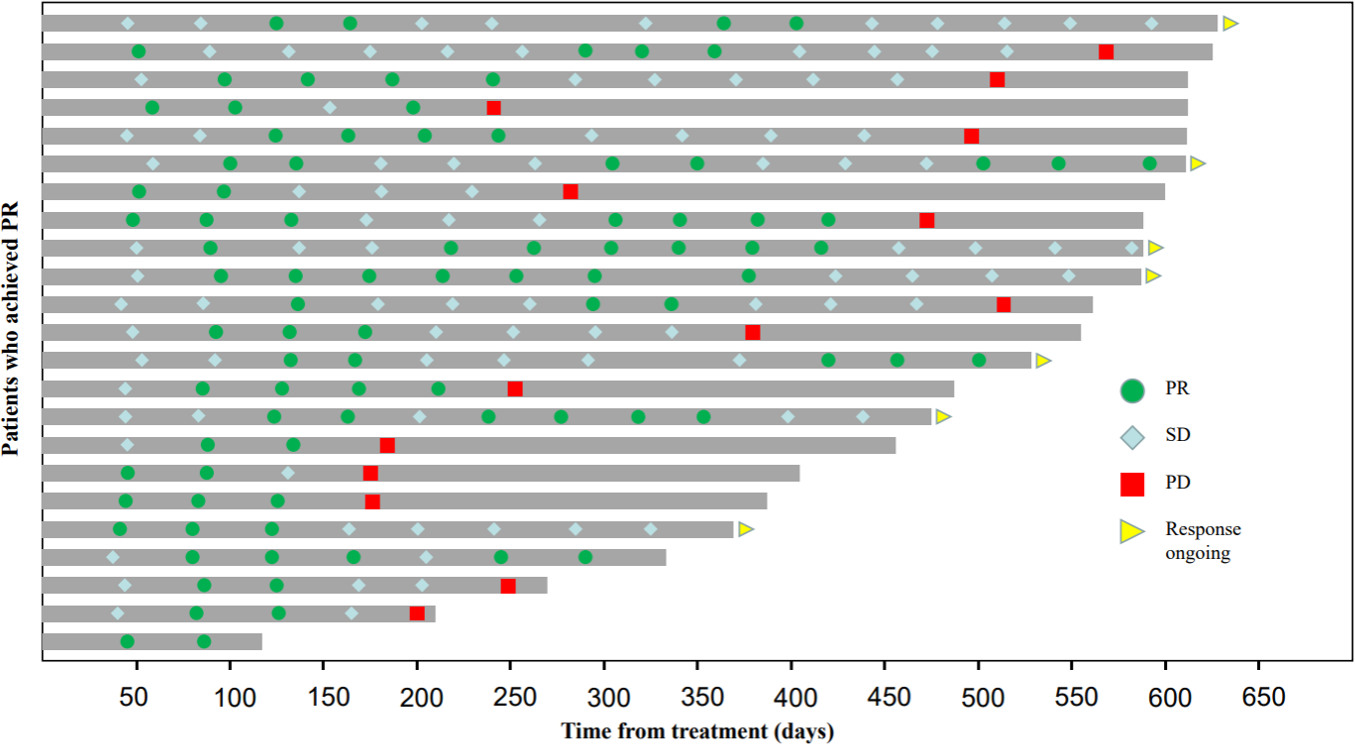
Swimmers plot of time on treatment for patients who achieved PR.

**Figure 4:**
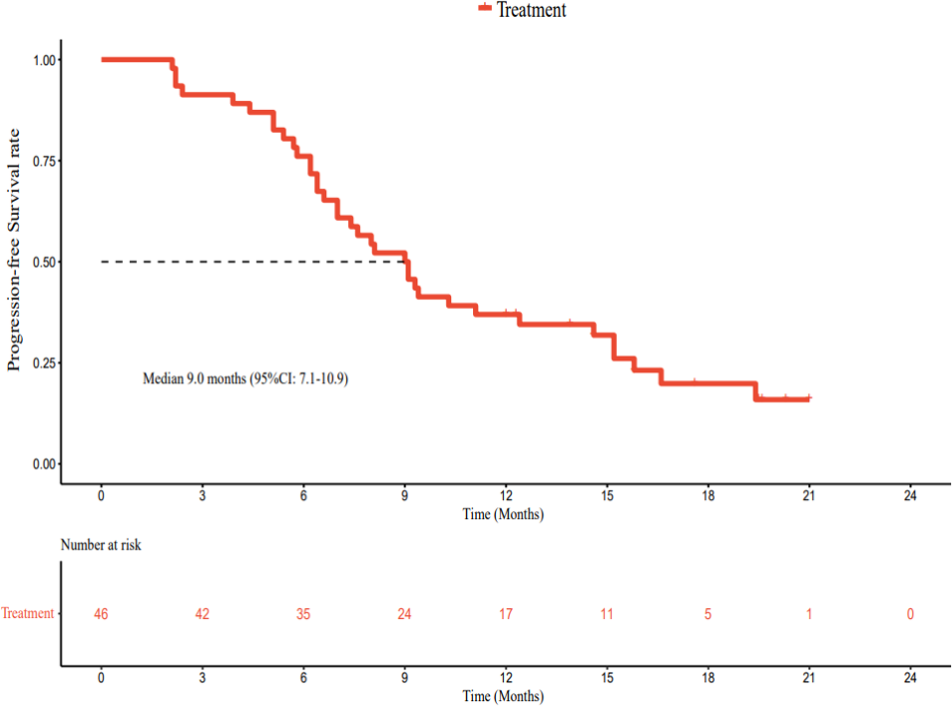
Kaplan–Meier plot of progression-free survival.

**Figure 5:**
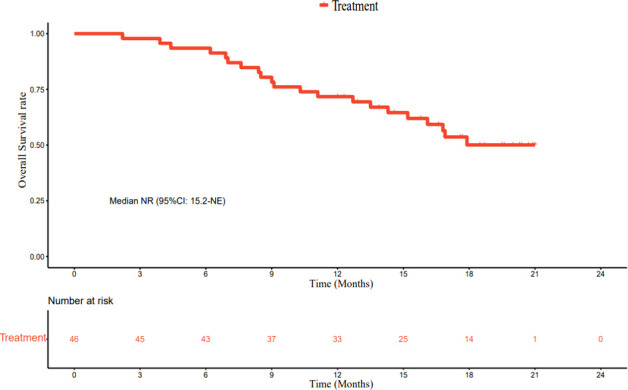
Kaplan–Meier plot of overall survival.

As of March 1, 2022, the median follow-up time was 18.7 months (95% CI: 17.2–20.2 months). In accordance with the analysis based on the RECIST v1.1 guidelines, in terms of the best overall response, there were 0 cases of CR, 23 cases (50.0%) of PR, 19 cases (41.3%) of SD, and 4 cases (8.7%) of progressive disease (PD). The ORR was 50.0% (95% CI: 34.9–65.1%) and the DCR was 91.3% (95% CI: 79.2–97.6%) (**Table 2**). The best percentage change in the target size is shown in **Figure 2**. The median DoR was 10.8 months (95% CI: 4.8–16.8 months). Of the 23 patients with PR, 7 (30.4%) continued to undergo treatment (**Figure 3**). The median PFS was 9.0 months (95% CI: 7.1–10.9 months) (**Figure 4**). At data cut-off, 21 patients had died, and 25 patients survived. The maturity of the data was 45.7% (21/46); thus, the median OS was not reached (**Figure 5**), and the OS at 6 and 12 months was 91.3% (95% CI: 79.2–97.6%) and 71.7% (95% CI: 56.5–84.0%), respectively.

**Table 2:** Tumor response.

Best overall response	Sintilimab+apatinib+capecitabine (N = 46)
Complete response (CR)	0
Partial response (PR)	23 (50.0%)
Stable disease (SD)	19 (41.3%)
Progressive disease (PD)	4 (8.7%)
**ORR, % (95% CI)**	50.0% (34.9-65.1%)
**DCR, % (95% CI)**	91.3% (79.2-97.6%)

### Safety

In terms of safety, the two cycles of the combination of the three drugs were completed in 46 cases, four cycles were completed in 42 cases, and immunotherapy was maintained in 17 cases for 1 year. All patients experienced treatment-related adverse events (TRAE). The most common TRAE were hand-and-foot syndrome (n = 37, 80.4%), abdominal pain (n = 32, 69.6%), nausea (n = 24, 52.2%), diarrhea (n = 21, 45.7%), fatigue (n = 19, 41.3%), hypertension (n = 13, 28.3%), hemoglobin decrease (n = 10, 21.7%), leukopenia (n = 7, 15.2%), elevated glutamic pyruvic transaminase (n = 6, 13%), thrombocytopenia (n = 5, 10.9%), itching (n = 5, 10.9%), and proteinuria (n = 5, 10.9%), but most of them were grade 1–2 TRAE. Grade 3–4 TRAE that occurred with an incidence of > 10% were hand-and-foot syndrome (n = 9, 19.6%) and hypertension (n = 5, 10.9%) (**Table 3**). TRAE necessitated an adjustment of capecitabine dose in 16 patients (34.8%), cessation of capecitabine treatment in 7 patients (15.2%), adjustment of the apatinib dose in 13 patients (28.3%), and cessation of apatinib treatment in 4 patients (8.7%). During the study, no patients died of TRAE, while 2 patients died of emergency AE during treatment (sudden rupture of the esophagus and gastric varices, resulting in massive bleeding).

**Table 3:** Treatment-related adverse events.

TRAE	Sintilimab+apatinib+capecitabine (N = 46)	
	Grade 1/2, n (%)	Grade 3/4, n (%)
Hand and foot syndrome	28 (60.9)	9 (19.6)
Abdominal pain	31 (67.4)	1 (2.2)
Diarrhoea	20 (43.5)	1 (2.2)
Nausea	24 (52.2)	0
Vomiting	11 (23.9)	0
Tiredness	19 (41.3)	0
Fever	4 (8.7)	0
Hypothyroidism	7 (15.2)	0
Hyperthyroidism	1 (2.2)	0
Cough	1 (2.2)	0
Hemoglobin decrease	8 (17.4)	2 (4.3)
Leukopenia	6 (13.0)	1 (2.2)
Neutropenia	4 (8.7)	0
Thrombocytopenia	5 (10.9)	0
Alanine aminotransferase increased	6 (13.0)	0
Aspartate aminotransferase increased	4 (8.7)	0
Rash	4 (8.7)	0
Pruritus	5 (10.9)	0
Bilirubin conjugated increased	3 (6.5)	0
hypertension	8 (17.4)	5 (10.9)
Albuminuria	4 (8.7)	1 (2.2)
Elevated serum creatinine	2 (4.3)	0
Hyperglycemia	1 (2.2)	0
Hypokalemia	1 (2.2)	0
Hyponatremia	2 (4.3)	0

TRAE, treatment-related adverse events; No grade 5 TRAE occurred.

### Exploratory analysis: PD-L1 expression

**Figure 6:**
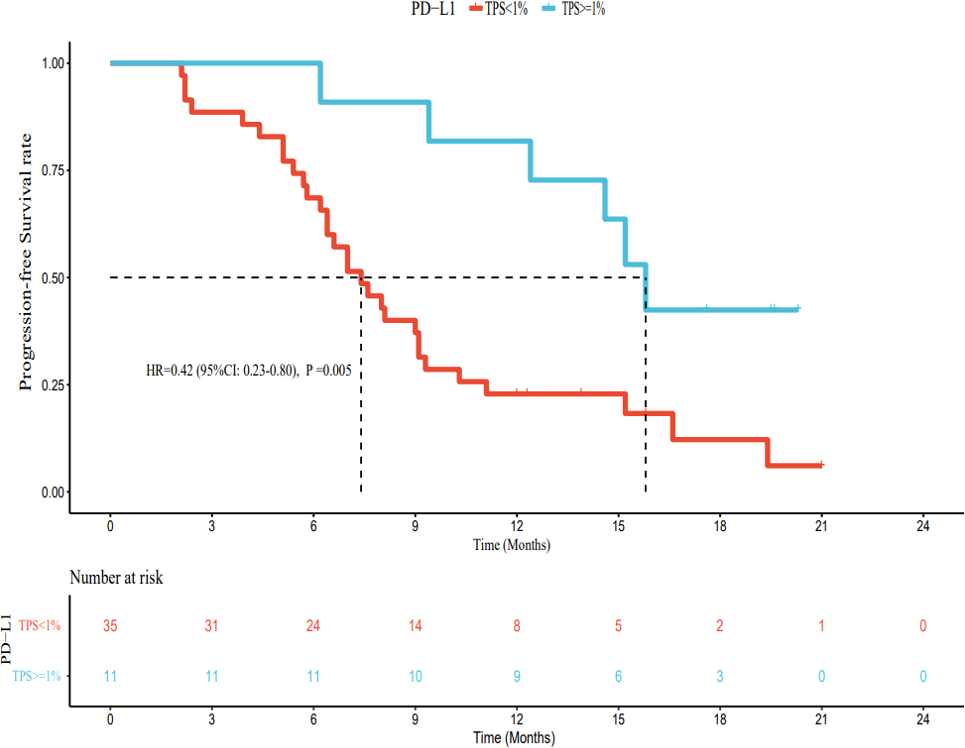
The relationship between PD-L1 expression and progression-free survival.

**Figure 7:**
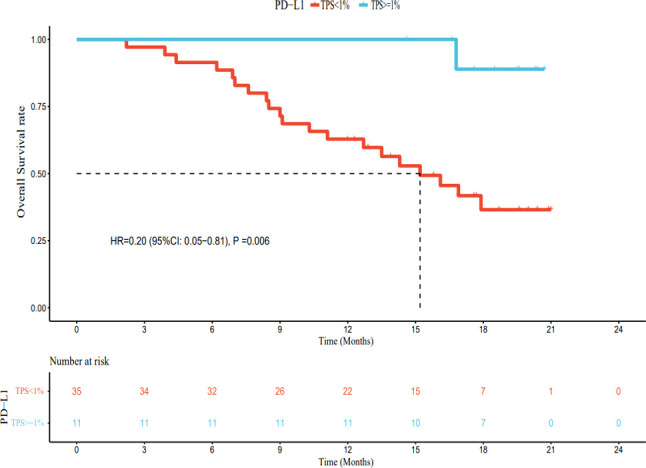
The relationship between PD-L1 expression and overall survival.

As of March 1, 2022, of the patients with PD-L1 TPS < 1%, 30 had PD, 5 had no disease progression, 20 had died, and 15 were still alive. The median PFS was 7.4 months (95% CI: 5.8–9.0 months), and the median OS was 15.2 months (95% CI: 11.2–19.2 months). Among patients with PD-L1 TPS ≥ 1%, 6 had developed PD, 5 still had no disease progression, 1 had died, and 10 survived. The median PFS was 15.8 months (95% CI: 14.1–17.5 months). The median OS was not reached. The median PFS and OS of patients with PD-L1 TPS ≥ 1% were significantly longer (hazard ratio [HR] 0.42, 95% CI: 0.23–0.80; HR 0.20, 95% CI: 0.05–0.81) than those for patients with PD-L1 TPS < 1%; this difference was statistically significant (**Figures 6**, **7**). Multivariate analysis showed that PD-L1 TPS expression was an independent prognostic indicator of PFS (**Table 4**).

**Table 4:** Cox regression analysis of PFS.

Variables	Univariate Analysis			Multivariate Analysis		
	HR	95% *CI*	*P*	HR	95% *CI*	*P*
Age(years)						
≤56	1					
>56	1.15	0.60-2.24	0.67			
Gender						
Female	1					
Male	0.75	0.31-1.81	0.52			
ECOG PS						
0	1					
1	3.13	1.45-6.76	0.004	2.33	1.02-5.32	0.045
BCLC						
B	1					
C	1.37	0.42-4.50	0.60			
Pathological differentiation						
III	1					
IV	0.83	0.36-1.93	0.67			
Unknown	1.91	0.39-9.30	0.42			
HBV infection						
Yes	1					
No	0.68	0.34-1.36	0.28			
Child-Pugh class						
A	1					
B	2.29	1.02-5.14	0.045	2.03	0.88-4.68	0.098
Extrahepatic spread						
Yes	1					
No	0.88	0.41-1.88	0.74			
Portal vein invasion						
Yes	1					
No	0.39	0.20-0.78	0.008	0.61	0.29-1.27	0.185
AFP, ng/ml						
<400	1					
≥400	1.60	0.83-3.09	0.16			
PD-L1 TPS						
<1%	1					
≥1%	0.42	0.23-0.80	0.005	0.32	0.13-0.79	0.014

## Discussion

Unresectable HCC is still challenging to treat because of its aggressiveness, high recurrence and metastasis rates, and lack of effective anti-tumor drugs. Our study was a single-arm, phase II clinical study that reached the pre-set main endpoint. The ORR of the blind independent image evaluation was 50.0%, and the DCR was 91.3%. In contrast, per the IMbrave150 study, the ORR and DCR of atezolizumab combined with bevacizumab (T+A), which has approved by the FDA as a first-line treatment, were 24.6% and 70%, respectively, in the Chinese population (30). In addition, in published and ongoing phase II clinical trials, the ORR of other first-line combination therapies ranged from 29.4% to 46% (12, 14, 31, 32). The regimens used in these trials were immunotherapy combined with anti-angiogenesis or immunotherapy combined with chemotherapy, while our study regimen was a combination of immunotherapy, anti-angiogenesis, and chemotherapy. The ORR of the primary endpoint in our study was higher than that in other similar studies, indicating that our study achieved significant anti-tumor effects and provided strong evidence for a hypothesized synergistic effect between immunotherapy, anti-angiogenesis treatment, and chemotherapy.

In terms of response duration, the median PFS in our study was 9.0 months, numerically superior to the 6.8 months reported by the T+A combination (15), the 5.5 months reported for sintilimab plus bevacizumab biosimilar (16), and the 5.7 months reported by the RESCUE study (12). It was similar to the 9.3-month PFS published in the KEYNOTE-524 study (32). Our secondary endpoint, the median OS, was not reached; this was related to an insufficient follow-up time. Up to the data cut-off point (March 1, 2022), the median follow-up time was 18.7 months. Only 21 patients had achieved the endpoint (death); 25 patients survived, and the data maturity was 45.7% (21/46). Therefore, further follow-up is required.

In terms of safety, our study continued the administration of other immunotherapy-based combination therapies with no specific TRAE. Most patients had grade 1/2 TRAE, while only 28.3% of patients had grade 3/4 TRAE, relatively lower than that reported by the IMbrave150 (15) or other studies (12, 14) and slightly higher than that reported by the AK105-203 study (33). More than 10% of grade 3/4 TRAE were associated with toxicity related to apatinib and capecitabine, characterized by hand-and-foot syndrome and hypertension, resulting in 15.2% and 8.7% of patients discontinuing capecitabine and apatinib, respectively. After the dose reduction or apatinib and/or capecitabine withdrawal, the AE was relieved or disappeared; thus, safety was controllable. The incidence of irAE was low, and no patients had grade 3 or higher irAE. During the study period, no patient died of TRAE, and only two patients died of massive bleeding due to sudden rupture of esophagogastric varices.

PD-L1 expression in tumor cells is detected using immunohistochemical methods and specific antibodies. In general, the higher the PD-L1 expression, the more likely patients are to benefit from immunotherapy. For example, in non-small cell lung cancer, if the expression of PD-L1 is more than 50%, the therapeutic effect on the patient will be better (34). Currently, the NCCN guidelines for non-small cell lung cancer recommend the detection of PD-L1 status before first-line immunotherapy. There is no need to detect PD-L1 status in the second-line treatment using nivolumab and atezolizumab, while second-line therapy with pembrolizumab can be used in the combined treatment of patients with PD-L1 TPS ≥ 1%, and first-line monotherapy is approved for patients with advanced non-small cell lung cancer with PD-L1 TPS ≥ 50% (35). However, in patients with unresectable HCC, the relationship between the expression of PD-L1 and the efficacy of immune checkpoint inhibitors is unclear (36). Therefore, we explored the relationship between the expression of PD-L1 and drug efficacy. Our results showed that patients with PD-L1 TPS ≥ 1% had significantly longer median PFS and OS than those with PD-L1 TPS < 1%, and this difference was statistically significant. However, in the present study, the number of patients in the PD-L1 TPS ≥ 1% group was small (only 11 cases), and most patients had a PD-L1 TPS < 1%. Thus, our results need to be validated by larger studies with a longer follow-up period.

As an early clinical trial with a single-center and single-arm design, the relatively small sample size was a limitation of our study. Second, only 63% of the patients in our study were infected with the hepatitis B virus, compared to the proportion of liver cancer patients in the Chinese population, i.e., 80% (33). Therefore, the results should be verified in studies using a larger sample size. In addition, the long-term survival data obtained in our study require further follow-up.

In conclusion, sintilimab combined with apatinib plus capecitabine has good safety and anti-tumor activity as a first-line treatment for unresectable HCC. This treatment approach may be an ideal new treatment strategy for unresectable HCC; our results indicate that further multi-center, prospective, randomized, large-sample clinical studies are warranted.

## Data availability statement

The original contributions presented in the study are included in the article/Supplementary Material. Further inquiries can be directed to the corresponding author.

## Ethics statement

This study was reviewed and approved by The Evaluation Committee of Medical Ethics institutions of Yichang Central people’s Hospital. The patients/participants provided their written informed consent to participate in this study.

## Author contributions

Research conception and design: XX. Patients were screened and follow-up data were collected: DLL, LX, JJ, DB, ZC, JH, YZ, YQ, DJL, YP, and SL. Data analysis: DLL, LX. Writing manuscripts: DLL and LX. Review manuscript: XX. All authors contributed to the manuscript and approved the submitted version.

## Funding

This study was funded by the Clinical Research Special Fund of Wu Jieping Medical Foundation (No: 320.6750.19079), CSCO Cancer Immunotherapy Research Fund (No: Y-XD2019-030), and the Major Project of Hubei Provincial Health and Family Planning Commission (No: WJ2017Z026).

## Conflict of interest

The authors declare that the research was conducted in the absence of any commercial or financial relationships that could be construed as a potential conflict of interest.

## Publisher’s note

All claims expressed in this article are solely those of the authors and do not necessarily represent those of their affiliated organizations, or those of the publisher, the editors and the reviewers. Any product that may be evaluated in this article, or claim that may be made by its manufacturer, is not guaranteed or endorsed by the publisher.
